# Corneal collagen crosslinking in patients treated with dextran versus isotonic hydroxypropyl methylcellulose (HPMC) riboflavin solution: a retrospective analysis

**DOI:** 10.1186/s40662-018-0116-z

**Published:** 2018-09-10

**Authors:** Patrick B. Rapuano, Priya M. Mathews, George J. Florakis, Stephen L. Trokel, Leejee H. Suh

**Affiliations:** Department of Ophthalmology, Edward S. Harkness Eye Institute, Columbia College of Physicians and Surgeons, 635 West 165th Street, New York, NY 10032 USA

**Keywords:** Corneal crosslinking, Isotonic HPMC, Dextran

## Abstract

**Background:**

Corneal collagen crosslinking (CXL) is a widely used treatment for halting the progression of keratoconus. Although initial studies of CXL were performed with a riboflavin solution containing dextran, recent protocols for CXL have indicated the use of a riboflavin solution containing isotonic hydroxypropyl methylcellulose (HPMC). This study was performed to investigate differences in visual outcomes and Scheimpflug (Pentacam) analysis in patients who have undergone epithelium-off CXL with riboflavin solution containing either 20% dextran versus 1.1% HPMC.

**Methods:**

All patients in this non-randomized, non-masked, retrospective cohort analysis were treated at Edward S. Harkness Eye Institute, Columbia University Medical Center, New York, NY, USA. Thirty-seven eyes of 33 patients were crosslinked with a dextran solution and 19 eyes of 19 patients crosslinked with an isotonic HPMC solution, both using an epithelium-off 30-min, 3 mW/cm^2^ protocol. All patients had a diagnosis of keratoconus or post-refractive surgery ectasia. Best spectacle corrected visual acuity (BSCVA) and Pentacam parameters were compared at all follow up visits (1, 6, 12, and 24 months). Differences between groups treated with HPMC and dextran were compared using student’s t-test. Differences between treated eye and fellow eye were calculated and compared between HPMC and dextran groups using paired t-test.

**Results:**

Patients treated with a dextran solution had significantly greater improvement in BSCVA at 1, 6, and 24 months (*p* < 0.05) compared to the isotonic HPMC-treated group. Kmax increased in both groups at 1 month; however, HPMC-treated patients had a greater increase compared to dextran-treated patients (*p* = 0.01). Kmax decreased in both groups at 6 and 12 months, although this finding was only significant in the HPMC-treated group at 12 months.

**Conclusions:**

Our data suggest that crosslinking with the dextran solution may result in significantly better visual outcomes (demonstrated by visual acuity) compared to the isotonic HPMC riboflavin solution. Dextran solutions may have other potential advantages intrinsic to its biochemical properties facilitating more efficient crosslinking. Further research and long-term evidence regarding the use of dextran versus HPMC riboflavin solutions in collagen crosslinking is necessary.

## Background

Keratoconus (KCN) is a condition characterized by thinning of the corneal stroma and progressive deformation of the cornea to a conical shape. Corneal collagen crosslinking (CXL) is an evolving therapy shown to be effective in halting the progression of keratoconus and post-refractive surgery ectasia [1–7].

The CXL procedure consists of photosensitizing the cornea with a riboflavin solution and irradiating the cornea with UVA light [1]. CXL to stop the progression of KCN and post-refractive surgery ectasia has recently been approved by the United States Food and Drug Administration (FDA) using a 0.154% riboflavin in 20% dextran solution.

One obstacle in evaluating the potential efficacy of CXL is the variation in composition of the riboflavin solutions that are in use. Both HPMC- and dextran-based riboflavin solutions are widely used with a recent preference being shown for HPMC solutions, possibly because they do not cause thinning of the corneal stroma [8]. Although many of the early trials showing the efficacy of CXL were performed with a dextran-based riboflavin solution, more recent studies frequently use HPMC-based riboflavin solutions. Although there are proposed benefits of CXL with HPMC, there is a dearth of published data providing justification for the switch in common use from dextran to HPMC.

Proposed benefits of an HPMC-based riboflavin solution compared to a dextran-based solution include absence of intraoperative corneal thinning [8–11], increased diffusion rate [12], and convenience of use [10, 13]. These advantages primarily focus on intraoperative effects of HPMC-based and dextran-based riboflavin solutions. However, to our knowledge, there has been no published data comparing clinical outcomes between CXL with different isotonic solutions.

This study is a retrospective cohort analysis comparing clinical outcomes in patients with progressive keratoconus or post-refractive surgery ectasia who were treated with CXL with a dextran-based or HPMC-based riboflavin solution. All other treatment variables were identical.

## Methods

The study protocol was approved by the Columbia University Institutional Review Board in accordance with the Declaration of Helsinki and was Health Insurance and Portability Accountability Act (HIPAA) compliant. Study subjects completed the study procedure between September 2010 and August 2015.

### Study subjects

Eligible subjects were 18 years of age or older and signed a written informed consent. Subjects with a diagnosis of keratoconus had one or more of the following: (1) presence of central or inferior steepening on the Pentacam tomography map (Oculus Inc., Arlington, WA, United States), (2) axial topography consistent with keratoconus, (3) presence of Fleischer ring, Vogt striae, corneal thinning, or corneal scarring. Progressive disease defined by one of the following in the past 24 months or less: (1) increase of > 1 diopter (D) in the steepest keratometry value (Kmax) or astigmatism evaluated by subjective manifest refraction, (2) documented decrease in visual acuity associated with irregular astigmatism.

Patients with a diagnosis of post-refractive surgery ectasia had disease defined by history of keratorefractive surgery and two of the following: (1) steepening on corneal topography, (2) thinning of the cornea, (3) shift in position of the thinnest portion of the cornea, (4) development of myopic astigmatism, (5) development of irregular astigmatism, (6) loss of BSCVA.

Contact lens wearers were required to remove contact lenses prior to the screening refraction: 3 days for soft lenses, 1 week for soft extended wear, 2 weeks for soft toric lenses, and 2 weeks for rigid gas permeable lenses.

Patients were excluded from the study if they met any of the following criteria: (1) ocular condition in eyes treated by CXL that could require additional treatment and predispose the eye to complications, (2) clinically significant corneal scarring unrelated to CXL, (3) chemical injury to the eye treated by CXL, (4) patients with a current condition that interferes with or prolongs epithelial healing, (5) previous CXL treatment in either eye (fellow eye was not excluded if first eye was crosslinked as part of this study).

The subjects were recruited at The Edward S. Harkness Eye Institute at Columbia University in New York, NY. Thirty-seven eyes of 33 patients were crosslinked with MedioCROSS® riboflavin/dextran solution from September 2010 to January 2014 (Avedro, Inc., Waltham, MA, United States). Nineteen eyes of 19 patients were crosslinked with Peschke® M riboflavin/HPMC solution from January 2014 to August 2015 (Peschke Trade, Hunenberg, Switzerland). This transition to an HPMC-based solution was made due to a reported increase in penetration of riboflavin into the corneal stroma with the HPMC-based solution and the ease of use of this less viscous solution^10^.

### Intervention

This study employed an epithelium-off 30-min UVA exposure of 3 mW/cm^2^ after stromal saturation with either riboflavin solution. All subjects were treated at The Edward S. Harkness Eye Institute at Columbia University in New York, NY by one of three surgeons (GF, LS, ST).

An approximately 9 mm diameter epithelial debridement was performed with an Amoils brush (Innovative Excimer Solutions, Ontario, Canada). Subjects in the dextran-treated group were treated with a MedioCROSS® 0.1% riboflavin, 20% dextran 500 solution. Subjects in the HPMC-treated solution were treated with a Peschke® M solution containing 0.1% riboflavin and 1.1% HPMC. Riboflavin solutions were then applied every 3 min for 30 min. Subsequently, all subjects were treated for 30 min with a UV-X™ Version 1000 light source from IROC with 365 nm UVA light with a beam diameter of 9 mm and an irradiance of 3 mW/cm^2^ (IROC Innocross AG, Zug, Switzerland). The respective riboflavin solutions were applied every 3 min during the 30 min of UV light exposure. Intraoperatively, the cornea was maintained at a thickness > 400 μm by measuring pachymetry at multiple timepoints. If found to be < 400 μm, a hypotonic HPMC-based riboflavin solution was applied to swell the cornea to > 400 μm.

Patients were managed post-operatively with placement of a bandage soft contact lens with topical antibiotics and topical steroids. The bandage contact lens was removed and the topical antibiotic discontinued when the epithelium was fully healed. Topical steroids were tapered off over a course of 1 month.

### Evaluations

Pre-op baseline exam was performed as well as follow-up eye exams at 1 week, 1 month, 3 months, 6 months, and every 6 months thereafter consisted of: (1) uncorrected distance visual acuity (UCVA) (2) best spectacle-corrected visual acuity (BSCVA), (3) manifest refraction, (4) Pentacam tomography measurements, (5) intraocular pressure, and (6) slit lamp exam of cornea, anterior chamber, and lens.

### Data collection

A spreadsheet was made from the data collected from examinations during a retrospective chart review. Chart review was individually performed by two of the authors (PR and PM), and all discordant values were compared and agreed upon for the final dataset. All Pentacam scans were reviewed for every patient to ensure adequate Pentacam reported data quality. When multiple scans were available for a single visit, the scan with the best Pentacam reported data quality was selected. When multiple reliable scans or exclusively unreliable scans were available for a single visit, the scan with the median Kmax value was selected for each eye.

### Statistical analysis

Group differences in baseline characteristics were assessed using the student’s *t*-test for normally-distributed continuous variables and chi-squared test for categorical variables. The main outcome variables of interest (BSCVA, Kmax) at 1, 6, 12, and 24 months were compared in the eyes treated with dextran versus those treated with HPMC using student’s *t-*test. The difference in BSCVA and Kmax between the treated eye and fellow eye was calculated for both the dextran and HPMC groups, and were compared in the dextran versus HPMC group using paired *t*-test.

## Results

A total of 56 eyes of 51 patients underwent CXL between September 2010 and August 2015. Thirty-seven eyes of 33 patients were treated with a dextran-based riboflavin solution, and 19 eyes of 19 patients were treated with an HPMC-based riboflavin solution.

Baseline characteristics of the dextran and HPMC groups are summarized in Table 1. There were no significant differences in baseline or demographic characteristics between the treatment groups (*p* > 0.05 for all). Data comparing treatment groups are reported in Table 2 and data comparing each group to fellow eye controls are reported in Table 3.

**Table 1:** Baseline characteristics of subjects by treatment group

	HPMC-treated	Dextran-treated	*p*
	(*n* = 19)	(*n* = 37)	
Age, years	26.7 ± 8.2	30.8 ± 10.3	0.12
Male, *n* (%)	15 (78.9)	29 (78.4)	0.63
Post-Lasik Ectasia, *n* (%)	1 (5.2)	9 (24.3)	0.08
Keratoconus, *n* (%)	18 (94.8)	28 (75.7)	0.08
BCVA preoperative (treated eye), logMAR	0.21 ± 0.17	0.31 ± 0.24	0.11
BCVA preoperative (control eye), logMAR	0.10 ± 0.16	0.07 ± 0.16	0.45
Kmax preoperative (treated eye), diopters	61.1 ± 8.7	57.7 ± 8.1	0.17
Kmax preoperative (control eye), diopters	51.0 ± 3.7	51.0 ± 6.7	0.98

*BCVA=* best corrected visual acuity, *HPMC=* hydroxypropyl methylcellulose

**Table 2:** Comparison of BSCVA and Kmax between HPMC vs. Dextran-treated Groups

	HPMC Group		Dextran Group		
	Mean	*n*-value	Mean	*n*-value	*p*-value
Change in BSCVA (SD)					
**1 Month**	**0.16 (0.15)**	**17**	**−0.05 (0.20)**	**30**	**< 0.001**
**6 Months**	**0.00 (0.17)**	**15**	**− 0.13 (0.17)**	**27**	**< 0.05**
12 Months	−0.03 (0.16)	15	−0.16 (0.22)	20	0.07
**24 Months**	**−0.01 (0.13)**	**9**	**−0.18 (0.17)**	**15**	**< 0.05**
Change in Kmax (SD)					
**1 Month**	**3.32 (4.89)**	**11**	**0.12 (2.23)**	**25**	**< 0.05**
6 Months	−0.20 (2.22)	12	−1.29 (3.12)	24	0.29
12 Months	−0.45 (2.35)	11	−2.41 (6.21)	16	0.33
24 Months	−1.82 (3.38)	9	−1.45 (2.65)	11	0.78

bolded values represent statistical significance

*BSCVA=* best spectacle corrected visual acuity, *HPMC=* hydroxypropyl methylcellulose, *SD=* standard deviation

**Table 3:** Changes in BSCVA and Kmax between Treatment Groups vs. Fellow Eye Controls

HPMC	Treatment Eye	Control Eye		
Mean Change in BSCVA (SD)	Mean	Mean	*n* value**	*p*-value
**1 Month**	**0.18 (0.17)**	**0.01 (0.06)**	**12**	**< 0.05**
6 Months	− 0.02 (0.16)	− 0.04 (0.09)	14	0.72
12 Months	− 0.05 (0.15)	− 0.02 (0.08)	14	0.65
24 Months	− 0.03 (0.13)	− 0.03 (0.08)	8	0.99
Mean Change in Kmax (SD)				
1 Month	4.9 (5.89)	−0.40 (2.93)	6	0.08
6 Months	−0.27 (2.13)	0.81 (1.14)	9	0.13
**12 Months**	**−0.88 (2.18)**	**1.13 (1.74)**	**9**	**< 0.05**
Dextran	Treatment Eye	Control Eye		
Mean Change in BSCVA (SD)	Mean	Mean	*n* value	*p*-value
1 Month	−0.03 (0.20)	− 0.02 (0.11)	26	0.74
**6 Months**	**−0.13 (0.18)**	**0.00 (0.08)**	**22**	**< 0.01**
**12 Months**	**−0.16 (0.22)**	**0.00 (0.10)**	**19**	**< 0.01**
**24 Months**	**−0.19 (0.17)**	**0.00 (0.08)**	**10**	**< 0.01**
Mean Change in Kmax (SD)				
1 Month	0.34 (1.88)	0.39 (0.81)	16	0.95
6 Months	− 1.25 (3.67)	0.24 (0.96)	16	0.17
12 Months	−2.19 (6.84)	−0.42 (1.09)	13	0.38

bolded values represent statistical significance

**number of patients is less than Table 2 due to incomplete data availability

*BSCVA=* best spectacle corrected visual acuity, *HPMC=* hydroxypropyl methylcellulose, *SD=* standard deviation

There were significant differences in log of the minimum angle of resolution (logMAR) BSCVA in the dextran-treated group compared with the HPMC-treated group at 1 month (− 0.05 vs. 0.16, *p* < 0.001, *n* = 30 vs. 17), 6 months (− 0.13 vs. 0.00, *p* < 0.05, *n* = 27 vs. 15), and 24 months (− 0.18 vs. -0.01, *p* < 0.05, *n* = 15 vs. 9); the difference at 12 months was not statistically significant (− 0.16 vs. -0.03, *p* = 0.07, *n* = 20 vs. 15). The dextran-treated group showed a significant improvement in logMAR BSCVA compared to fellow eye controls at 6 months (− 0.13 vs. 0.00, *p* < 0.01, *n* = 22), 12 months (− 0.16 vs. 0.00, *p* < 0.01, *n* = 19), and 24 months (− 0.19 vs. 0.00, *p* < 0.01, *n* = 10). However, in the HPMC-treated group there was a significant decrease in BSCVA compared to fellow eye controls at 1 month (0.18 vs. 0.01, *p* < 0.01, *n* = 12), and no difference at 6 months (− 0.02 vs. -0.04, *p* = 0.72, *n* = 14), 12 months (− 0.05 vs. -0.02, *p* = 0.65, *n* = 14), or 24 months (− 0.03 vs. -0.03, *p* = 0.99, *n* = 8).

The dextran-treated group showed a significant decrease in Kmax compared to the HPMC-treated group at 1 month (0.12 vs. 3.32 diopter (D), *p* = 0.01, *n* = 25, 11); however, there was no significant difference at 6 months (− 1.29 vs. -0.20 D, *p* = 0.29, *n* = 24, 12), 12 months (− 2.41 vs. -0.45 D, *p* = 0.33, *n* = 16, 11), or 24 months (− 1.45 vs. -1.82, *p* = 0.78, *n* = 11, 9). There was no significant change in Kmax in the dextran-treated group when compared to fellow eye controls at 1 month (0.34 vs. 0.39, *p* = 0.95, *n* = 16), 6 months (− 1.25 vs. 0.24, *p* = 0.17, *n* = 16), or 12 months (− 2.19 vs. -0.42 D, *p* = 0.38, *n* = 13). There was no significant change in Kmax in the HPMC-treated group when compared to fellow eye controls at 1 month (4.9 vs. -0.4 D, *p* = 0.08, *n* = 6) or 6 months (− 0.27 vs. 0.81 D, *p* = 0.13, *n* = 9); however, there was a significant decrease in Kmax in the HPMC-treated group compared to fellow eye controls at 12 months (− 0.88 vs. 1.13 D, *p* = 0.02, *n* = 9).

This retrospective analysis of corneal crosslinking clinical outcomes showed better visual acuity results with a dextran-based riboflavin solution as compared to an HPMC-based riboflavin solution. BSCVA was improved by 1 month in the dextran-treated group and this improvement is seen also at 6 months and at 24 months, while mean BSCVA in the HPMC-treated group did not improve.

The dextran-treated group showed an advantage over the HPMC-treated group in measurement of Kmax at 1 month. The dextran-treated group had a stable Kmax at 1 month, whereas the HPMC-treated group had an increase in Kmax at 1 month, although this increase was not statistically significant when compared to fellow eye controls. There was no significant difference in the change in Kmax between the HPMC and dextran treatment groups at 6, 12, and 24 months. The HPMC group showed a statistically significant decrease in Kmax compared to fellow-eye controls at 12 months whereas the dextran group did not; however, this decrease in Kmax in the HPMC group did not correlate with visual improvement.

## Discussion

This introductory study is the first to demonstrate differences in visual outcomes up to 2 years after CXL between isotonic HPMC and dextran-based riboflavin solutions and provides justification for further investigation into the differences between CXL with different solutions. The improvements in visual acuity demonstrated in this study after CXL are consistent with the current literature, as a recent systematic review and meta-analysis has found improvements in BSCVA as early as 3 months after crosslinking [5]. Additionally, prior studies comparing visual outcomes after CXL between dextran-based riboflavin solutions and hypotonic HPMC-based riboflavin solutions have seen a statistically significant improvement in vision with dextran compared to hypotonic HPMC at 1 year [14]. These data show that although Kmax results after CXL are comparable with these solutions, the visual outcomes are better with dextran solutions than with HPMC solutions. Further investigation with more data is required to better understand the connection between BSCVA and Kmax in patients after CXL.

One potential explanation for the difference in outcomes is a differential penetration of UV light into the corneal stroma between the two solutions. It has been shown that dextran-based riboflavin solutions significantly thin the cornea and isotonic HPMC-based riboflavin solutions have little impact on corneal thickness and may slightly swell or slightly thin the cornea during CXL [10, 11]. Recent studies have shown that a 20% dextran 0.1% riboflavin solution with a 30-min induction period allows UV light to penetrate a greater percentage of the corneal stroma than does a 1.1% HPMC 0.1% riboflavin solution even with a 10-min induction [15]. The difference in the depth of penetration only increases when the induction time for the HPMC-based riboflavin solution was increased to 30 min. In addition to allowing for a greater percentage depth of UV light penetration, the transient thinning of the cornea with the use of 20% dextran is likely to result in a significantly greater depth of post-operative cornea treated with UV light once the dextran-thinned cornea returns to its pre-operative thickness.

The depth of penetration of the UV light will likely correlate with the demarcation line visible after CXL. The demarcation line has been suggested as an objective marker to determine the efficacy of corneal crosslinking and increasing depth of demarcation line has been associated with improved Kmax outcomes [16]. In contrast, others in the crosslinking field question the paradigm of “the deeper, the better” regarding demarcation line [17]. A recent study reporting on demarcation line depth in contact lens-assisted CXL finds a deeper demarcation line with isotonic 1.1% HPMC than with the standard 20% dextran solution, although these authors do not assert that a deeper demarcation line represents a desirable outcome [18].

In the future, we would like to see isotonic HPMC and dextran-based riboflavin solutions compared in a large prospective randomized trial to determine whether the differences in clinical outcomes are truly clinically significant. Ideally, we would have anterior segment optical coherence tomography (OCT) data for these patients in order to compare the depth of the demarcation line in the two treatment groups to determine whether a deeper demarcation line is associated with improved clinical outcomes. Additionally, in future studies we would have Pentacam densitometry data in order to formally grade stromal haze and to correlate postoperative haze and visual acuity after CXL with HPMC and dextran-based riboflavin solutions.

There are some limitations to our retrospective study. First, there is a small number of patients in each group, which decreases by the first year of follow up. The small number of patients limited our ability to compare differences in crosslinking outcomes between patients with keratoconus and post-refractive surgery ectasia. However, we performed our study in a controlled environment with the same treatment and follow-up protocol. Although the number of patients is relatively small, we believe that the results of our study justify the need for higher-powered studies in the future. Secondly, keratoconus is a bilateral disease, therefore the “control” eye likely has a degree of corneal ectasia as well, which may affect the results. However, we believe that it is important to compare the treated eye with the fellow eye to demonstrate halting the disease. Thirdly, we have included patients with KCN and post-LASIK ectasia in this study, which are two distinct disease processes, and analyzed the results of CXL in these patients in a single group. Finally, this study does not include anterior OCT data or Pentacam densitometry data for correlation of stromal haze with visual acuity. We hope future CXL trials will look more closely at this relationship given the differences in visual acuity shown in this study.

## Conclusions

In conclusion, this study is the first to describe differences in outcomes of epithelium-off corneal collagen crosslinking between different isotonic riboflavin solutions. In the past, many studies have looked at differences in UV exposure times and method of entry for riboflavin solutions into the corneal stroma. By effectively comparing the different isotonic riboflavin solutions, a more efficient method of epithelium-off corneal collagen crosslinking can be determined for future treatments.

## Abbreviations

BSCVA: Best spectacle corrected visual acuity
CXL: Corneal collagen crosslinking
FDA: United States Food and Drug Administration
HIPAA: Health Insurance and Portability Accountability Act
HPMC: Hydroxypropyl methylcellulose
KCN: Keratoconus
Kmax: Steepest keratometry value
LogMAR: Log of the minimum angle of resolution
OCT: Optical coherence tomography
UCVA: Uncorrected distance visual acuity
